# Pheromone Autodetection: Evidence and Implications

**DOI:** 10.3390/insects7020017

**Published:** 2016-04-25

**Authors:** Robert Holdcraft, Cesar Rodriguez-Saona, Lukasz L. Stelinski

**Affiliations:** 1 Marucci Center for Blueberry and Cranberry Research and Extension, Rutgers University, 125A Lake Oswego Road, Chatsworth, NJ 08019, USA; crodriguez@aesop.rutgers.edu; 2 Citrus Research and Education Center, University of Florida, 700 Experiment Station Road, Lake Alfred, FL 33850, USA; stelinski@ufl.edu

**Keywords:** pheromone autodetection, anosmia, electroantennogram, single-sensillum recording, pheromone-binding-protein, intra-sexual communication, plume competition, mating disruption

## Abstract

Olfactory communication research with insects utilizing sex pheromones has focused on the effects of pheromones on signal receivers. Early pheromone detection studies using the silkworm moth, *Bombyx mori* L., and Saturniids led to the assumption that emitters, especially females, are unable to detect their own pheromone. Pheromone anosmia, *i.e.*, the inability of females to detect their conspecific sex pheromone, was often assumed, and initially little attention was paid to female behaviors that may result from autodetection, *i.e.*, the ability of females to detect their sex pheromone. Detection of conspecific pheromone plumes from nearby females may provide information to improve chances of mating success and progeny survival. Since the first documented example in 1972, numerous occurrences of autodetection have been observed and verified in field and laboratory studies. We summarize here a significant portion of research relating to autodetection. Electrophysiological and behavioral investigations, as well as expression patterns of proteins involved in pheromone autodetection are included. We discuss problems inherent in defining a boundary between sex and aggregation pheromones considering the occurrence of autodetection, and summarize hypothesized selection pressures favoring autodetection. Importance of including autodetection studies in future work is emphasized by complications arising from a lack of knowledge combined with expanding the use of pheromones in agriculture.

## 1. Introduction

### 1.1. Background

In the process of describing autodetection, and why it is imperative to expand our understanding of it, some general background concepts should be reviewed. In discussing insect pheromone-mediated sexual communication, long-range pheromone signals can be grouped variously: by which sex produces them, *i.e.*, exclusively by male, female, or both sexes; or grouped by which sex responds to the signal. Generally accepted groups are therefore female- or male-produced sex-pheromones that are detected by and attract exclusively the opposite sex, and female- and/or male-produced aggregation pheromones that are detected by and attract both sexes. Sex pheromones, usually considered to be for the sole purpose of attracting a mate of the opposite sex, are particularly common among the Lepidoptera, and the Coleopteran family Scarabidae. Among both groups, it is frequently (though not always) the female that calls and the male who responds. Aggregation pheromones, found frequently among other families of the Coleoptera, are not as easily delineated as they can be emitted by individuals or groups of either or both sexes, but in all cases result in the attraction of both. Aggregation is also thought to serve multiple functions beyond mate location, such as host plant location or to overcome host defenses, though mating likely remains an important result.

**Figure 1:**
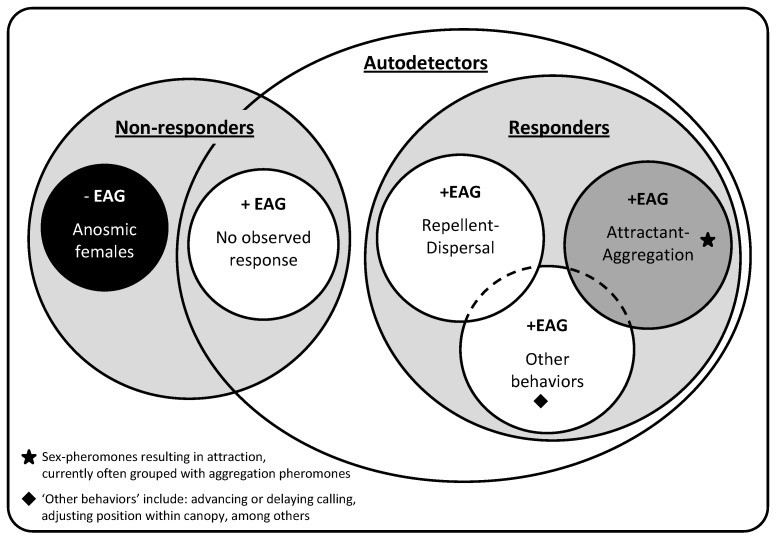
Venn diagram of behavioral (repellence, attraction and dispersal, and aggregation) and electrophysiological (electroantennogram (EAG)) responses, and no responses, of females to conspecific-produced sex pheromones.

Complications to the above methods for grouping pheromones arise when it is discovered that for some species, traditionally considered to use sex pheromones, members of the same sex as the caller can detect their conspecific pheromone. Such complications have occurred in cases among both the Lepidoptera and Scarabidae, particularly, though not exclusively, among species with female-produced pheromones. In most of these cases, it had been assumed that while males could detect the pheromones, females could not. There is, however, growing evidence that this assumption is not always true. In some cases the pheromone only attracts males, thus operating as a sex pheromone, but may have other behavioral effects on females; for example, a repellent effect, or causing an advance or delay in calling initiation. Additionally, in some rare cases, the pheromone attracts both sexes, thus operating more like an aggregation pheromone (Figure 1). One conundrum is how to categorize a species’ pheromone if it is found to attract both sexes when the pheromones of related species only attract males.

These complications have occurred with sufficient frequency to cause the adoption of the term “autodetection” to describe pheromone-producers that can detect conspecific pheromone, particularly regarding female-produced pheromones. It has been suggested that usage of the term “autodetection” to describe the cases thus far observed may be somewhat flawed, as it suggests individuals are “self-detecting”, as opposed to using conspecific pheromone cues as a form of intrasexual communication. Despite its possible inaccuracy, the term has been readily adopted in discussions regarding the above-described phenomenon. The majority of research on autodetection focuses on Lepidoptera with fewer studies on Coleoptera. Results from electrophysiological work using electroantennograms (EAG) of both male and female moths have provided the basis for the creation and clarification of the terms “pheromone anosmia” and “autodetection”. These are almost entirely used in reference to female-produced sex pheromones of moths, and the response, or lack thereof, from female antennae exposed to those pheromones. “Female antennal anosmia” is defined as the inability of female antennae to detect their conspecific sex pheromone, while “female autodetection” occurs when females can detect their sex pheromone (Figure 1). As noted above, the behavior of female insects that can detect their own pheromone signal can sometimes be similar to the response mediated by aggregation pheromones (Figure 1); however, female behaviors are influenced in many ways (described below) as a result of autodetection.

Additional clarification of the above definitions will likely arise from the expanding work focusing on pheromone-binding proteins (PBP) and pheromone-receptor proteins (PR). These two separate protein groups are crucial in the process of pheromone detection and are readily found in the pheromone receptor-cells of male antennae. Both PBP and PR have been investigated in female antennae as well, and this work has begun to provide some evidence that pheromone autodetection may not be a result of the absence or presence of these proteins in antennal receptors so much as a function of their levels in those receptors. Within this review, we provide a first attempt at analyzing the relationship between quantitative estimates of PBP/PR in female antennae and resulting levels of electrophysiological response to pheromone.

### 1.2. History of Anosmia vs. Autodetection

Since the first simple tests of sex attractants by Fabre in 1879 using the great peacock moth, *Saturnia pyri* (Denis and Schiffermüller) (Lepidoptera: Saturniidae), the Saturniid moths with their large antennae have been test subjects in sex-attraction studies. Indeed, they are still commonly referenced in entomology courses and textbooks when sex pheromones are discussed. The obvious sexual dimorphism of their antennae helps to convey the mechanisms and sensitivity of pheromone reception. The similarly endowed gypsy moth, *Lymantria dispar* (Linnaeus) (Lepidoptera: Erebidae), has also been an important model system since the inability of the females to fly helps conceptualize the role that sex pheromones play in division of risk and parental investment [1]. However, after the identification of the silkworm moth pheromone “bombykol” in the late 1950s [2] and the concurrent development of the electroantennogram (EAG) [3,4], the silkworm moth, *Bombyx mori* (Linnaeus) (Lepidoptera: Bombycidae), became an established model organism for pheromone research. Well-established rearing methods for silkworm moths likely played a role in the selection of *B.* *mori* as a research subject, as they provided researchers with the large numbers of adult females needed for pheromone gland extraction. Additionally, the large antennae of males of that species likely helped in the development of the EAG by providing antennae that were easy to manipulate. Given that female antennae did not show EAG responses to bombykol, compared to obvious responses from males, the EAG became a standard method to assay male response to female-produced sex pheromones [3,4,5]. The choice of *B. mori* as a model organism contributed to identification of insect pheromones through development of behavioral and electrophysiological methods, but may have led to an initial assumption that most female moths cannot perceive their own pheromones. This assumption was pervasive. For example, Schneider *et al.* [6] stated that “Female antennae of most moth species are apparently anosmic to their own odour, while autodetection of female pheromones is a less frequently observed phenomenon”.

It was almost two decades after the initial ground breaking advances in insect sex pheromone research of the 1950s that the unpredicted behavior of virgin females of the noctuid moth, *Trichoplusia ni* (Hübner) (Lepidoptera: Noctuidae), was observed; namely, their capture in significant numbers, along with males, in pheromone-baited traps [7]. This observation provided a clue that long-distance pheromone-mediated interactions within some species were not always limited to calling females and responding males. Indeed, two possible causes for such behavior by *T. ni* females were suggested: male-produced sex pheromones [8,9] or that *T. ni* females were detecting and responding to the synthetic female sex pheromones used to bait the traps [10]. These more complex interactions have yet to be fully explored.

## 2. Types of Evidence

### 2.1. Criterion for Selecting Literature

To locate and select articles for inclusion in this review of the existing evidence for autodetection thus far, an initial literature search was undertaken using the following search engines: Rutgers University Libraries literature search tool, BioOne, CAB Abstracts, Google Scholar, IngentaConnect, JSTOR, Mendeley, PubMed, SpringerLink, and Web of Science. To cast as wide a net as possible, key search terms included an array of applicable subjects and combinations/variations thereof. A partial list of such terms included but was not limited to: “autodetection”, “pheromone autodetection”, “pheromone anosmia”, “female sex pheromone”, “electroantennagram/graph”, “EAG”, “single sensillum recording”, “SSR”, “female electroantennagram/EAG”, “female insect sex pheromone detection”, “female insect pheromone trap capture”, “female response to conspecific sex pheromone”, “pheromone binding protein”, “pheromone receptor protein”, amongst others. This initial search was as inclusive as was feasible in an attempt to locate as many papers as possible that might touch on the subject of autodetection.

The term “autodetection”, when found in the literature, was almost exclusively applied to long-range female-produced sex pheromones, with only two known cases where autodetection was discussed but not directly applied to male-produced compounds [6,11]. Most occurrences of the term were within the Lepidoptera (excluding butterflies) and the Coleopteran family Scarabidae; both groups contain a plethora of published works identifying various species female-produced sex pheromones. Partly due to this innate bias, we limited our review to only those species with female-produced sex pheromones, leaving out male-produced pheromones. This choice was also partially due to the fact that in most, if not all cases, long-range male-produced pheromones result in attraction of both sexes, which make male sex pheromones difficult to distinguish from aggregation pheromones. References to groups commonly found to use aggregation pheromones, particularly male-produced as is often case in the Coleopteran families Curculionidae and Cerambycidae, were excluded entirely. This was done to avoid the redundancy inherent in the phrase “autodetection of aggregation pheromones”, *i.e.*, the ability of a pheromone emitter to detect its own pheromone; although considered novel in the case of autodetection, it is inherent in the very definition of aggregation pheromones.

Full scanning of all remaining papers was required since many did not mention inclusion of females in their titles, and several failed to mention it even in abstracts. After scanning was complete, only those studies that provided some type of data regarding female autodetection remained. Data on female electrophysiological responses to pheromones, and/or detection of pheromone binding and receptor proteins in females, came from sources as variable as side notes in male focused papers to studies dedicated specifically to investigation of these attributes in females. Data sources for female behavioral responses to pheromones were similarly variable, with many dedicated lab studies focused solely on females, but a large portion of available field data coming from side notes or anecdotes about females caught unexpectedly in pheromone-baited traps intended to capture males.

### 2.2. Sexual Dimorphism of Antennae

To convey the concept of differential pheromone detection abilities between sexes, entry level entomology courses and textbooks include comparisons of gross morphological differences in antennal size and complexity [12,13]. While it is true that extreme sexual dimorphism exits among the Saturniidae, and could be described as having predictive value for general olfactory sensitivity, the morphological differences are not as extreme among many other families such as the Tortricidae and Noctuidae [14]. As a result, gross morphology fails to provide the level of sensitivity required to define the patterns of occurrence of more subtle traits such as pheromone autodetection that can vary between species with little difference in antennal sexual dimorphism. Studies of antennae comparing morphology and pheromone sensitivity among various types of sensilla have provided evidence that gross morphology does not directly link with sex pheromone detection [15,16].

As a result, we decided to abandon the use of antennal morphology as evidence for autodetection, despite its long history of inclusion in textbook discussions of pheromone detection, focusing instead on direct electro-physiological evidence. The majority of studies included in this review focus on two major areas in pheromone communication relevant to autodetection: (1) detection ability; and (2) resulting behaviors. From electrophysiological and behavioral studies, we were able to classify 45 species in the order Lepidoptera, nine in the Coleoptera, two in the Blattodea, and one in the Diptera as either autodetectors or anosmic (Table 1 and Table 2).

### 2.3. Behavioral Observations

We found 42 reports using the term “autodetection”, or otherwise dealing with the effects of autodetection on insect behavior (Table 1). Of these, 28 cases (67%) involved species in the order Lepidoptera, 12 (29%) in the Coleoptera, one (2%) in the Blattodea, and one (2%) in the Diptera. While behavioral studies seem well represented among the Lepidoptera and scarab beetles, less than a third of them (27%) are from field data (including both field observations and unexpected captures of females in pheromone traps). The lack of dedicated field studies investigating behavior changes from autodetection can be attributed in large part to the difficulty of observing and possibly subtle differences in behaviors under field conditions. Low light levels during the natural calling periods of many species are one example of hurtles that face a researcher desiring to perform detailed observations of behavior in the field. The remaining 73% of behavior studies included in Table 1 are various types of laboratory assays. The increasing prevalence of studies using observation chambers, multi-choice olfactometers, and flight mills have contributed to our understanding of the majority of effects that autodetection has on pre- and post-mating behaviors.

The effects of autodetection may be subtle as to make their documentation via behavioral observation difficult. The possibility exists that detection of a conspecific pheromone may act as a primer signal, resulting in internal physiological changes that would be unapparent to even the most dedicated observer. Physiological effects of autodetection could impact hormonal activity, mating receptivity, the rate of pheromone synthesis, or the component ratios of pheromone blends. The difficulties inherent in documenting such subtle effects do not preclude them from investigation, as is described below, but it does increase the likelihood of false negative or false positive results.

There is a high likelihood that a significant number of “failed” attempts at documenting behavioral effects of autodetection have gone unpublished, and were unable to be included in our review. Therefore, it would be improper to draw conclusions based on the percentage of behavioral studies resulting in verified changes. To further illustrate this point, of those behavioral studies published (Table 1), there were 52 distinct results (including multiple studies per species, and multiple species per study). Of those, 43 showed autodetection, two were unclear, and only seven suggested anosmia. Furthermore, of the seven “negative” results of investigations, only three were published independently of an accompanying positive result (behavioral or physiological). This suggests that results showing anosmia may be unlikely to be published. This could be a bias against “negative results”. Given this possible bias, we have refrained from using Table 1 to make predictions about possible frequencies of behavioral effects among autodetectors. The remainder of this section lists the various behavioral effects that have been confirmed through positive observations or experimental proof, grouped into the three sub-classes within the “responders” described in Figure 1.

Eight lepidopterans displayed increased movement in the presence of pheromone; suggesting females may be repelled or are triggered to disperse. Females of the tortricid, *Choristoneura fumiferana* (Clemens), increased dispersal following pheromone pre-exposure [17,18]. Females of two noctuids, *Heliothis armigera* (Hübner) and *Helicoverpa zea* (Boddie) were repelled by their pheromone to a significant degree in olfactometer tests [19], while females of a third noctuid, *Spodoptera littoralis* (Boisduval) experienced reduced mating and increased flight activity [20]. Females of the sesiid, *Vitacea polistiformis* (Harris), increased local movements altering its calling position during exposure [21]. Females of the pyralid, *Ephestia kuehniella* (Zeller), were repelled, dispersing from an area permeated with pheromone-baited traps [22]. A recent study using a flight mill [23] showed increased movement of both *Grapholita molesta* (Busck) and *Choristoneura rosaceana* (Harris) after exposure to pheromone. These behaviors may contribute to fitness of the various species. Computer models suggest repellent behavior and/or movement away from other pheromone sources may increase the likelihood of female mating [24], and dispersal triggered by high local levels of pheromone could drive mated females from areas of high population density, reducing resource competition among progeny [22].

Fourteen lepidopteran species adjusted calling behavior in various ways (see references [25,26,27,28,29,30,31,32,33,34,35,36,37,38,39,40,41,42] in Table 1). Two tortricids, *G. molesta* and *C. fumiferana* advanced the onset of calling up to 2 hours. Several tortricids, *Adoxophyes orana* (Fischer von Röslerstamm), *Adoxophyes honmai* (Yasuda), *Homona magnanima* (Diakonoff), *Argyrotaenia velutinana* (Walker); one noctuid, *Spodoptera exigua* (Hübner); and the sesiid *V. polistiformis*, delayed calling, while *C. rosaceana* displayed a variety of changes. The tortricid and arctiid moths, *Cydia pomonella* (Linnaeus) and *Utetheisa ornatrix* (Linnaeus) respectively, increased calling frequency and intensity, while the noctuid moth, *Pseudaletia adultera* (Schaus) called more and extended the calling window. *Spodoptera littoralis* (Boisduval) extended calling late into scotophase. *Cydia fagiglandana* (Zeller), *Cydia splendana* (Hübner), and *Utetheisa ornatrix* (Linnaeus), two tortricids and an arctiid respectively, synchronized calling, a response similar to “female pheromonal chorusing”, suggesting novel communal adaptations that increase group attractiveness.

Kuhns *et al.* [28] found reduced mating success after exposure to pheromone in both *G. molesta* and *Pandemis pyrusana* (Kearfott), though no changes to calling behaviors were observed. Stelinski *et al.* [23], however, found no effect of pheromone pre-exposure on mating success of either *C. rosaceana* or *G. molesta*. These contradictory results from separate studies with *G. molesta* highlight variation of behavioral responses depending possibly on assay conditions. Three tortricids (*Eupoecilia ambiguella* (Hübner), *Pandemis pyrusana* (Kearfott), and *Lobesia botrana* (Denis and Schiffermüller)) and an arctiid (*Euplagia quadripunctaria* (Poda)) displayed no observable behavioral responses when exposed to pheromone (Refs [25,26,27,28,29,30,31,32,33,34,35,36,37,38,39,40,41,42] in Table 1).

Autodetection resulting in attraction or aggregation may be more easily observed than the behaviors listed above, and has often been discovered during field trials when pheromone-baited traps serendipitously capture significant numbers of females. One of the earliest reports suggesting autodetection was capture of *T. ni* females in monitoring traps [7] and the behavior was later verified with further field trapping [10]. Similar attraction behavior is assumed to have resulted in increased larval density near pheromone traps targeting the sesiid, *Melittia cucurbitae* (Harris), because of increased density of females near these sources of pheromone [42]. So far only one lepidopteran species has verified attraction to conspecific pheromone in the lab. Gravid females of the noctuid moth, *Sesamia nonagrioides* (Lefebvre), were attracted to pheromone sources in dual-choice olfactometer assays [36].

Behavioral evidence of autodetection among the Scarabidae (order: Coleoptera) (Refs [43,44,45,46,47,48,49,50,51,52,53,54] in Table 1) was similarly observed given attraction or aggregation to pheromone sources. Females of *Anomala orientalis* (Waterhouse) [43], *Anomala rufocuprea* (Motschulsky) [45,46], *Cotinis nitida* (Linnaeus) [47,48], *Holotrichia consanguinea* (Blanchard) [49], and *Maladera (matrida) insanabilis* (Brenske) [53,54] were all caught in traps baited with female-produced sex pheromones, originally thought to be only attractive to males. Attraction to pheromone sources was later confirmed by field observations for three of these species: *A. orientalis* [44], *H. consanguinea* [49], and *M. insanabilis* [53]. Grouped females of two additional scarabs, *Holotrichia loochooana* *loochooana* (Sawada), and *Holotrichia serrata* (Fabricius) were observed calling simultaneously in the field [50,52], which may be an aggregative behavior. Yarden and Shani [53] found female *M.* *insanabilis* in pheromone traps and then confirmed female attraction to conspecific pheromone with lab-based olfactometers. In addition, Yasui *et al.* [51] verified attraction of *H.* *loochooana* *loochooana* females to calling females and pheromone lures.

Beyond Lepidoptera and Coleoptera, there were only two species wherein female behaviors, in response to female-produced sex pheromones, were reported in our literature review (Table 1). Baker and Longhurst [56] reported captures of female olive fruit fly, *Bactrocera (Dacus) oleae* (Rossi) (Diptera: Tephritidae) in pheromone-baited traps; however, attraction to visual cues could have played a role. It has been shown in olfactometer studies done by Ross and Tignor [55] that German cockroach, *Blattella germanica* (Linnaeus) (Blatodea: Blatellidae), females respond to the pheromone produced by female conspecifics.

### 2.4. Electroantennogram (EAG) and Single-Sensillum Recording (SSR)

To date, EAG/SSR of female antennae have been examined in 11 families of Lepidoptera, of which, seven families (Tortricidae, Noctuidae, Arctiidae, Cossidae, Sesiidae, Yponomeuta, and Pyralidae) exhibited positive responses in at least one, and often multiple species within a given family (Table 2). It should be noted that in two species, the presence of a response was reported in the most recent studies of those species but not in earlier works. Overall, these results suggest that, contrary to earlier assumptions, it is not rare among the Lepidoptera for the antennae of females to detect components of the female sex pheromone.

There is a wide range of pheromone loading rates among the studies listed. Amounts tested ranged from picograms to milligrams of pheromone, with additional variation in quality from rough extracts to multiple component blends of synthetic pheromones. These variations combined with different reporting styles make exact comparison of antennal sensitivity difficult, but two general trends emerge. First, female antennae are much less sensitive than those of conspecific males, with reported detection thresholds 50–100× higher than males. Second, female antennal responses have lower amplitudes (EAG) or firing frequencies (SSR) than males at equivalent doses. Only three of the lepidopteran families listed lacked any response to their pheromones: the Saturniidae, Bombycidae, and Geometridae. The sole sphingid reported, the tobacco hornworm, *Manduca sexta* (Linnaeus), showed mixed results. In one study, *M.* *sexta* showed no EAG responses to the two major pheromone components, bombykal and *E*10,*E*12,*Z*14-hexadecatrienal [57], while in a second study an SSR response was reported to one of the minor pheromone components, *Z*11-hexadecenal [58].

Generally, pheromone sensitivity in females is substantially lower than that recorded in males, as is often noted in the electrophysiological studies included herein. Additionally, documentation of pheromone receptors on female antennae shows a similar relationship, with females exhibiting dramatically fewer than males. For these reasons there is an increased risk of low-level responses being lost in signal noise, and generalist olfactory receptor activity. Further complications can arise from generalist receptors, which can potentially be activated merely by the key functional groups of pheromone components or particularly high doses, such that specialist pheromone receptors may only account for a small portion of an overall response. While these risks and complications do not seem evident in SSR studies, identification of individual pheromone receptors does not provide the same level of support for conclusions about overall antennal pheromone sensitivity. As a result, EAGs are generally considered to be the most definitive tests for pheromone sensitivity of both sexes since they display the summed response of all receptors on a given antenna when activated by the pheromone.

For the non-lepidoptera reported in Table 2 [90,91,92,93,94,95,96,97], four species of scarab beetles, three from the genus *Anomala* and one *Holotrichia*, showed confirmed autodetection by females, as did the sole representative of the Blattodea. Similar to the lepidopterans, female autodetection responses to conspecific pheromone in all of these species were significantly lower than that of males at the same pheromone concentrations, with both lower amplitudes and higher detection thresholds occurring in every case.

### 2.5. Pheromone-Binding- and Pheromone-Receptor- Proteins in Female Antennae

Two separate protein groups, pheromone-binding proteins (PBP) and pheromone-receptor proteins (PR), abundant in the antennal receptor cells of males, are crucial in the process of pheromone detection [98,99], and have been investigated extensively. As inclusion of antennae from both sexes in such studies has become more frequent they have not only provided a greater understanding of the process of pheromone detection by males, but have created a means to expand understanding of autodetection by females.

In 1981, Vogt and Riddiford [100] first described antenna-specific PBPs found in male antennal tissues of three saturniids and one sphingid: *Antheraea pernyi* (Guerin Meneville), *A. polyphemus* (Cramer), *Hyalophora cecropia* (Linnaeus), and *M. sexta*, respectively. An apparent absence of PBP from female antennae, compared to their abundance in males, provided the basis for defining the activity of these previously undescribed proteins as male-specific pheromone binders. A contrast in PBP levels between highly sensitive males and anosmic females could be considered as physiological proof for the lack of autodetection.

With improvements in technology, the presence of PBPs and/or PRs have been detected in the antennal tissues of *B. mori*, *A. polyphemus* and *M. sexta* females, which were all previously considered to be anosmic [101,102,103,104]. Table 3 provides a summary of all species where PBP and PR expression in females has been investigated. Each of the nine families of Lepidoptera and Coleoptera examined to date has at least one species where these proteins (or precursors) have been detected. Indeed, PBP has been confirmed in females from all 26 species that have been tested for its presence, excluding *H. cecropia*, which has only been a subject of initial tests conducted in 1981 [100].

## 3. Relationships and Patterns

All evidence for autodetection in females is summarized in Table 4. The relative expression (F: M) of antennal detection proteins in female antennae, provided in detail in Table 3, were summarized in Table 4 by grouping them into the following categories: High (>66% to 100%), Moderate (>33% to 66%), Low (>0% to 33%), Not Detected (0%), Undetermined = Not Tested. Females showing EAG or SSR responses to pheromone have been assumed to have both PBP and PR present in their antennae for the purposes of this review. However, it is possible that “non-specialist” receptor neurons might also contribute to an EAG response to a pheromone component in the absence of PRs or PBPs. PBP has been detected in 25 out of the 26 species tested, supporting this assumption. When species included in either type of investigation are compared, even with the conservative assumption that absence of PBP is indicated by a lack of EAG/SSR response, 87% of the resulting 54 species still have presence of PBP confirmed. Considering that the Bombycidae and Saturniidae, two families that have never shown EAG/SSR responses, are positive for PBP, the above assumption is likely too conservative. The confirmed presence of PBP in the majority of females from this cross-section of moth species, including examples of both anosmia and autodetection, increases the likelihood that these proteins are present in the antennal receptor cells of females from most families of the Lepidoptera. Further survey of additional families and genera should continue to confirm or reject this tentative hypothesis that the presence of these proteins in female antennae may be ubiquitous in females of moth species. To quote Steinbrecht’s personal communication with Schneider: “If the Bombyx female does possess “autodetective” receptor-cells, pheromone anosmia (or conversely pheromone autodetection) in female moths would not be a qualitative but rather a quantitative sexual distinction” [6].

The presence or absence of autodetection would also likely be a function of the relative levels of PBP and PR proteins in female antennae. Further relationship between PBP/PR expression and antennal response (EAG/SSR) can be examined by going back through the literature summarized in Table 2 and Table 3. Out of the 67 species included in this review (Table 4), only 14 have usable data from both electrophysiological and protein expression studies of female antennae. Fourteen data points are not a very robust sample, and cannot provide a basis for strong conclusions, but they can provide a framework to build upon while revealing preliminary trends. Quantified estimates of protein levels and antennal responses were obtained from appropriate papers. The quantity and magnitude of the estimates were presented relative to their levels in male antennae *i.e.*, ratios (female:male) or percentages ((female/male) × 100). The validity of using characteristics of male antennae to represent the maximum potential for many parameters of pheromone detection should be acceptable since selection pressures of mate location would drive male characteristics toward their maximum. This provided a way to standardize parameter values when comparing multiple species. Standardization was considered desirable for two other reasons: (1) the difference in antennal sensitivity between sexes within a species may be less than that between the males or females of two different species and in such cases the relative sensitivities between males and females could be more uniform between species; and (2) the majority of papers listed in Table 2 and Table 3 reported female parameters as relative to males. Relative protein was almost always reported as a range (e.g., “levels in the female were 40%–50% that of the male”). The minimum and maximum values were used to calculate the average relative value.

**Figure 2:**
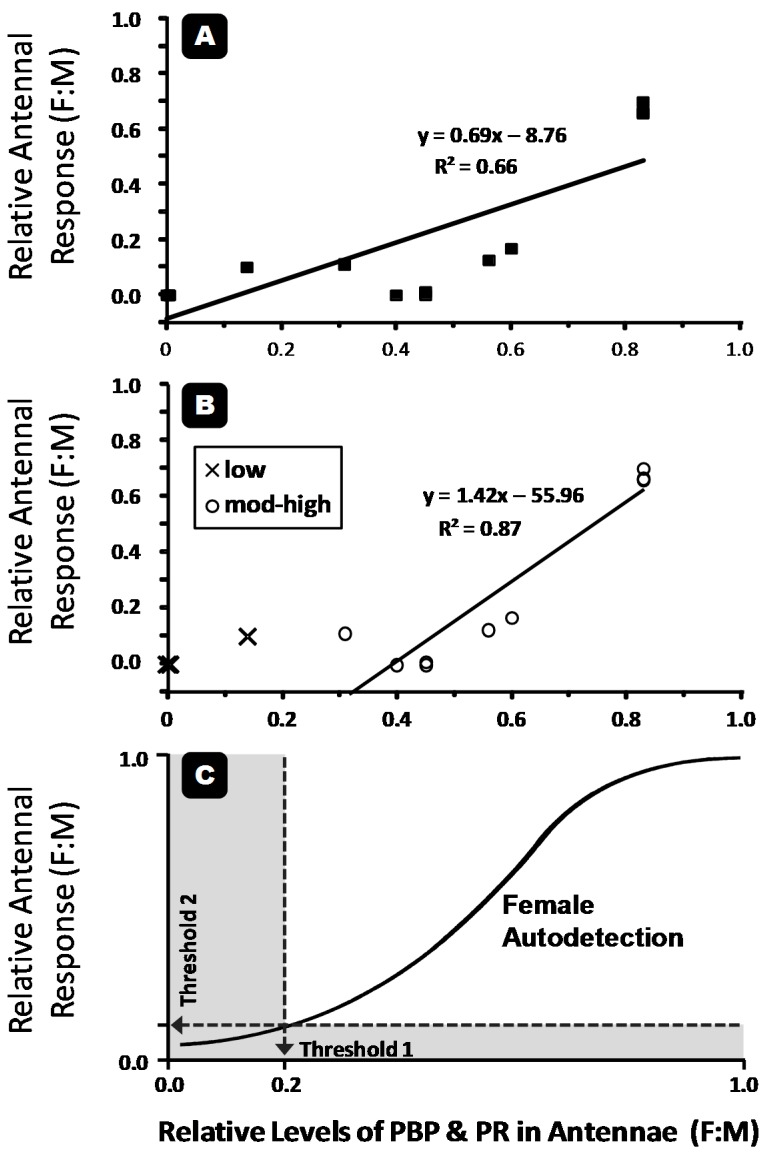
Relationships between relative levels of pheromone-binding (PBR) and pheromone-receptor (PR) proteins in female antennae of 14 species (see Table 4) and their relative antennal response: for all species (**A**); for species divided into two distinct groups based on levels of protein expression, a group that had little to no protein in their antennae (≤20% = “low”), and thus considered anosmic, and those that had higher levels of protein in their antennae (≥30% = “mod-high”), and thus considered here as autodetectors (**B**); and a theoretical relationship between relative levels of proteins in female antennae and their relative antennal response (**C**): species traditionally considered anosmic should fall in the gray areas below thresholds 1 and 2, while autodetecting females should fall somewhere along the linear portion of the curve.

The resulting correlations between protein level and antennal sensitivity (relative EAG amplitude in this case) are presented in Figure 2. The linear model in Figure 2A groups all 14 species and results in a low *R*^2^ value of 0.66. The model was then adjusted as is seen in Figure 2B; separating out five species with average relative protein expression less than 20% (low). The remaining nine species had levels of protein expression that ranged from 30% to 90% (high), and unsurprisingly, all turn out to be confirmed autodetectors (Table 3). The *R*^2^ value for this adjusted model improved significantly to 0.87 for the nine species of particular interest. Based on Figure 2B, all five species with protein levels below 20% could be considered anosmic. This division is a rough first approximation of a threshold for detection below which females are unresponsive, and above which they become more likely to autodetect. Figure 2D shows a theoretical relationship between relative levels of pheromone-binding or pheromone-receptor proteins in female antennae and relative antennal response. In this model, species traditionally considered anosmic should fall in the gray areas below either of two thresholds, while autodetecting females should fall somewhere on the linear portion of the curve. Although the linear models presented in Figure 2 should not be considered reliable until more species can be added (as they were only created with nine data points), they are presented as examples for predictions for future research.

## 4. Selection Pressures Favoring Autodetection

Unless a female gains some benefit from autodetection, the cost of producing pheromone-binding and receptor proteins should favor anosmia. Females can still gain some information about pheromone levels in their immediate vicinity without the level of sensitivity found in males. This lower sensitivity may balance the costs of maintaining the ability to autodetect with the potentially small or sporadic benefits gained by females from this additional information. The observed behavioral responses discussed in Section 2.2 match theoretical strategies that could improve chances of mating or progeny survival that have often been the subject of speculation [1,136,137,138,139]. Models of some behaviors have provided evidence of possible benefits [24]. The ability of females to autodetect, has also been proposed as a primitive precursor to dimorphic male sex pheromone systems in *S. littoralis* and *H. virescens* [16,140]. The physiological requirements for antennae to detect female pheromones, already in place in females with autodetection ability, could have allowed for an expansion of the role of female antennae into mate recognition. While not a direct benefit of autodetection, mate recognition and evaluation using male sex pheromones provides a benefit to females by preventing cross-species mating, and allowing them to determine suitable mates by pheromone quality. Overall, evidence from various areas of research lend support to the idea that beneficial behaviors could result from autodetection that would conserve resources, avoid competition, improve a female’s chances for successful mating, improve progeny survival, and even for avoiding predation.

### 4.1. Resource Limitation and Plume Competition

Pheromone production may be limited by available resources, especially in species where adults do little to no feeding. Harari *et al.* [139] described a cost of pheromone production and release that decreased a female’s total fecundity. If pheromone production utilizes limited resources that could otherwise be used for egg production, then behaviors that improve efficiency of pheromones for mate attraction should result in increased fitness. In cases where adult females do feed readily; however, pheromone production has not been shown to be resource limited [141]. Species that do limited feeding after maturation may therefore be more likely to exhibit behaviors resulting from autodetection that have this potential for improving mating efficiency.

The effects of “plume competition”, described as interference between the pheromone plumes of different females lowering mating success at high population densities, was modeled by Lundberg and Löfstedt [138]. They concluded that there should be selection pressure for “individually distinguishable pheromone plumes”, which may allow detection of competing plumes for avoidance—a beneficial trait. In the field, lowered mating success was found to occur in two separate lepidopteran forest pests species when they reached high densities [142,143]; the Eastern spruce budworm *C. fumiferana* (Lepidoptera: Tortricidae) and the Forest tent caterpillar, *Malacosoma disstria* (Hübner) (Lepidoptera: Lasiocampidae). Competition from upwind pheromone-baited traps interfering with downwind trap-catch was confirmed by Wall and Perry [144,145], further supporting this hypothesis. Similar plume competition among multiple pheromone emitters was documented by Miller *et al.* [146,147] where point-source based mating disruption was demonstrated to compete with actual females via false trail following. This type of competition was likely the major cause of reduced female mating success, demonstrating potential effects on female fitness. Behaviors observed to result from autodetection by females with the potential to avoid competition can be classified as either temporal adjustments (e.g., initiating calling earlier or extending calling later), or spatial adjustments (e.g., changing position/location locally or dispersing entirely from an area) (see references in Table 2 and Table 4). Many of these behaviors could potentially benefit females through avoidance of plume competition, or other types of competition for mates.

Similar selection pressures to those favoring distinguishable plumes would favor increased sensitivity in detection by either sex, possibly to the level of distinguishing among inter-individual variations in plume quality. The abilities of males to distinguish between variations in pheromone component ratios are well established and suggest that males already exhibit this level of sensitivity. This extreme level of sensitivity has not yet been demonstrated in females, though the EAG/SSR studies included in Table 2 do show that females have variable sensitivity to different pheromone components; no females were able to match male sensitivity to low pheromone dosages, *i.e.*, female detection thresholds were usually more than 50× higher than those of their conspecific males. Interestingly, antennal sensitivity to conspecific sex pheromone has been shown to increase with age in unmated virgin females of one of those same forest pests, *C. fumiferana* [61]. This suggests that for mature females, the benefits to be gained from improved antennal sensitivity can begin to outweigh the costs as the length of time spent unmated increases.

### 4.2. Cooperative and Dishonest Strategies in Aggregations

Aggregation and/or synchronization of calling could increase attractiveness of cooperating groups of females over longer ranges. Some cooperative behaviors resulting in highly attractive groups have been dubbed pheromonal chorusing by Lim and Greenfield [39,40], and have been suggested as one reason that scarab species aggregate in response to female sex pheromone [51,148]. A less cooperative strategy that could also result in attraction and apparent aggregations has been observed in some scarabs [51,149]. In those cases, “silent” females that avoid calling occur along with normal females in some aggregations. Once males locate an aggregation, they attempt to mate with any female they encounter regardless of type; silent females could conserve their own pheromone and gain increased fecundity by intercepting males called in by others. Females of a few scarab species have shown reductions in pheromone production and output with age potentially making them less attractive [150,151,152,153,154]. Such older females and with diminished resources would benefit tremendously by intercepting males.

An additional dishonest strategy that could provide “silent” females a completely separate benefit beyond conservation of resources could be at work as well. These females could benefit from a reduction in their risk of predation or parasitism by “illicit receivers” which are parasitoids and predators that eavesdrop on the mating signals of their prey to locate or track them. Predators have been shown to eavesdrop on both auditory signals [155,156] and olfactory signals [157,158,159,160,161] used by insects to locate mates. Specifically among the Lepidoptera, the male-produced sex pheromone of the greater waxmoth, *Galleria mellonella* (Linnaeus) (Lepidoptera: Pyralidae) is attractive to the parasitoid, *Bracon hebetor* (Say) (Hymenoptera: Braconidae) [159], while the female-produced sex pheromones of two tussock moth species, *Euproctis pseudoconspersa* (Strand) and *Euproctis taiwana* (Shiraki) (Lepidoptera: Lymantriidae) and two lepidopteran pests of olives, *Prays oleae* (Bernard) (Lepidoptera: Yponomeutidae) and *Palpita unionalis* (Hübner) (Lepidoptera: Pyralidae) were attractive to the Hymenopteran parasitoids *Telenomus euproctidis* (Wilcox) (Hymenoptera: Scelionidae) [160], and *Trichogramma oleae* (Voegelé and Pointel) (Hymenoptera: Trichogrammatidae) [161], respectively. The above cases are just a few examples of how predators and parasitoids exploit pheromone signals despite an assumed difficulty inherent in exploiting such specialized signals. Direct impacts on fitness from the increased predation risk represented by illicit receivers have been demonstrated in some cases [162,163]. Thus, two separate dishonest strategies can be at play: “silent” females can occupy satellite positions around a calling female to intercept arriving males while the caller bears the dual burdens of resource consumption and predation risk. To employ these dishonest strategies successfully, however, requires that the “cheaters” have an ability to detect and locate their “marks”, *i.e.*, the calling females that they will take advantage of, which is where autodetection plays a role.

### 4.3. Effect of Movement on Female Fecundity and Progeny Survival

The negative effects of aging on fitness of unmated females can be more direct than reductions in pheromone production. Reduced mating success and fecundity with delays in mating have been found for multiple species [164,165,166,167,168,169] and has been the subject of a recent meta-analysis that showed similar conclusions [170]. An ability to detect and avoid competitors could increase likelihood of mating early. Additionally, modeling work done by Pearson *et al.* [24] predicts improved mating success in mating disruption plots with increased female movement. This would mean that even if autodetection only stimulates an increase in random movement of unmated females their chances of mating, either early or late, may increase.

Post-mating competition for oviposition sites and progeny resources is another area in which autodetection could be beneficial. Oviposition-deterrent marking pheromones such that of the blueberry maggot fly, *Rhagoletis mendax* (Curran), deter females from laying more than one egg per berry. Communication between females in this case decreases competition that would be detrimental to progeny [171]. Similarly, sex pheromone detected near a potential host plant could indicate females are already present on that plant [172]. Avoidance of areas of high population density when searching for oviposition sites would reduce competition for resources among progeny. Dispersal behavior triggered by pheromone detection seems to occur with at least two lepidopterans. The tortricid moth, *C. fumiferana*, and the pyralid flour moth, *E. kuehniella*, both respond to pheromone-laden air with increased movement and dispersal post-mating [17,18,22].

## 5. Implications of Autodetection for Pheromone Use in Agriculture

### 5.1. Pheromone-Baited Monitoring Traps

Increasing use of pheromones as tools in agriculture and some of the unexpected subsequent behavior of females suggests this is a useful area of investigation. The use of pheromone-baited traps for monitoring insect pest populations has been thoroughly established as a crucial tool in Integrated Pest Management (IPM) worldwide [173,174]. Such traps are used extensively for estimating infestation and accurately timing insecticide applications. If, however, males and females are attracted to an area without the majority of them effectively captured and eliminated from the population, the presence of a trap could inadvertently increase the local rate of mating and oviposition. An influx from surrounding areas of many virgin or mated females could result directly in increased crop damage from the resulting larval infestation.

High levels of crop infestation by the squash vine borer, *Melittia cucurbitae* (Harris), were found in fields monitored with pheromone traps, while much lower infestations were found in nearby control fields without traps. This observation led to electrophysiological research that uncovered the ability of female *M*. *cucurbitae* to autodetect [42]. Though attraction was never proven, autodetection could hypothetically increase infestations associated with pheromone traps for certain species. However, such concerns are not generally realized as many insect species, particularly among the weevils (Coleoptera: Curculionidae), are monitored with aggregation pheromone-baited traps without resulting in subsequent infestations [175,176].

### 5.2. Pheromone Mating Disruption

The increased use of mating disruption for sustainable and non-toxic control of insect pest populations has added greatly to our reliance on pheromone-based technologies in agriculture. Continuing environmental and health concerns over the use of traditional insecticides will likely call for continued expansion of mating disruption to control more pest species [177] as will the introduction of transgenic crop plants possibly capable of producing and releasing pheromone [178]. However, mixed results from the application of mating disruption over the past few decades have highlighted aspects of pheromone communication that are incompletely understood, including female autodetection. Initial hypotheses regarding the failures of mating disruption focused almost exclusively on the effect of mating disruption on male behaviors [174,179]. Economic interest for the efficacy of this technology makes it important to complete our understanding of these complex behaviors and their possible effects. As a result, previously neglected subjects, such as the electrophysiological responses and behaviors of females are being included more frequently in new research on pheromone perception [177,180].

Mating disruption has shown mixed results particularly when applied to species that are known autodetectors [147]. Many behaviors exhibited by autodetectors have been hypothesized to help females avoid competition for mates as noted previously. Those same behaviors could account for that variation in the success of mating disruption since competition has proven to be a major mechanism of common methods of disruption [146,147,181]. As discussed previously, increases in local population density have been associated with attraction to pheromone-baited traps in at least one case. If attraction acts in disrupted fields in the same way, population density could increase to a point where random mating still takes place. It is also possible that aggregation of both sexes adjacent to disruption point sources could reduce overall disruption efficacy in a similar manner. Multiple mating pairs and individuals of the oriental beetle, *A. orientalis*, were found in close proximity to pheromone point sources in plots treated with pheromone for mating disruption [44]. As long as mated females do not move into disrupted fields, this type of aggregation should not reduce efficacy. Theoretical results of repellent reactions are dependent on whether virgin and mated females are affected similarly. If dispersive behavior is not common to both virgin and mated females, emigrating virgin females could conceivably mate beyond the disruptive border and return to oviposit in the unprotected crop. Only a small number of the studies that found repellent or dispersive behavior included mating status as a variable [17,18,19,21,22]. The results from those studies suggest that repellent behaviors are common to both virgin and mated females or are more likely after mating. In either scenario, there is little chance of a mated female returning to a disrupted area, but a reliable conclusion cannot be reached with such limited data. On the other hand, if both virgin and mated females are similarly repelled from disrupted fields, the effect is likely to improve disruption efficacy. However, this may be at the expense of neighboring farms, which could be infested with emigrating females and their resulting progeny.

## 6. Conclusions

The term “autodetection” may not be restricted exclusively to long-range female-produced sex pheromones; it could also apply to male-produced sex pheromones among Coleoptera. The term has been used in connection to male-produced hair-pencil and contact (aphrodisiac) pheromones [182,183,184,185], but male ability in this area is not surprising [1]. Male-produced pheromones that result in aggregations occur frequently in coleopteran families such as the Cerambycidae and Curculionidae [186,187,188,189,190,191,192,193,194,195]. While attraction/aggregation could result from males eavesdropping on each other’s sex pheromone signals, thus fitting the description of autodetection, such a result would be indistinguishable from male responses to aggregation pheromones. Additionally, observations with the Curculionidae suggest that pheromones produced by both sexes function to concentrate populations on suitable host plants [188,191,195] often in order to overwhelm host plant defenses [186]. In any case, it is evident that the term “autodetection” as currently defined applies most to detection of female-produced sex pheromone by conspecific females.

Herein, we reviewed 126 research papers that investigated some aspect of female autodetection among the Lepidoptera and the coleopteran family Scarabidae. Additionally, more than sixty papers have been incorporated that provide evidence supporting the possible theoretical benefits gained from autodetection. Research has focused, in large part, on species that damage crops or affect human health since development of technologies for agricultural and urban pest control provide an economic incentive for research, and this trend is not likely to change in the future. For example, adoption of mating disruption is likely to increase, as use of conventional pesticides is further restricted (US Environmental Protection Agency, Food Quality Protection Act) and as public demand for pesticide-free homes and produce rises [177]. However, mating disruption is a much more complex method of pest control than conventional insecticides and many factors remain undefined that might influence its success or failure.

Better understanding of the frequency and distribution of autodetection ability across the insect orders and families that use sex pheromones is needed, which can be gained by more thorough sampling, using a targeted phylogenetic approach. From the articles included in the electrophysiological and behavioral portions of this review, it is possible to confirm autodetection in 43 of 57 species in 11 of the 16 families thus far sampled (75.4% and 68.7%, respectively). A selection bias must certainly exist that favors inclusion of females suspected of such ability in these studies, so this cannot be considered a representative sample. However, many important pest species are included in this sample, and the clear majority displays this ability. For example, two lepidopteran families, the Tortricidae and Noctuidae, represent 46% of those species tested, and 22 of those 26 (84.6%) were confirmed autodetectors. In contrast, none of the six species from the family Saturniidae were found to autodetect. This single family represents only 10.5% of species tested (*n* = 57), but accounts for 42.9% of negative results (*n* = 14). The tortricid and noctuid families include some of the most important agricultural moth pests, while saturniids contain few if any. A positive trend between autodetection ability and pest-status thus far suggested may be invalid due to those same sampling biases, but its confirmation in over two thirds of the families and 75% of species tested thus far means the ability does not seem rare.

When female autodetection is included, modeling mating disruption mechanisms will increase in complexity. The various ways in which competing pheromone plumes from disruptors, monitoring traps, and actual females may interact, and how female behaviors might affect the results of disruption were discussed by Miller *et al.* [146]. They conclude that movement of female moths “is likely to diminish rather than enhance mating disruption” and that “disruption efficacy could be dramatically inferior to that predicted… if virgin females were repelled or deterred from calling”. The actual effects of various female behaviors on disruption remain largely untested, however. Once potential responses are defined and verified, predictions resulting from the improved models should be much stronger. The complex interactions collectively labeled “mating disruption” need to be further dissected. Improved models of disruption mechanisms could prove useful for evaluating this technology’s potential for control of various pests before expending money and time on field trials.

The high frequency of autodetection in moth families most often considered for control via mating disruption, and our lack of knowledge about how females respond to mating disruption practices is why further research is needed. When evaluating new applications of pheromone-based technologies, behavioral and electrophysiological studies should be done for both females and males along with methods for estimating essential antennal proteins like PBP and PR. In 1977, Birch [10] suggested that “the role of the sexes in response to pheromone should be investigated further, particularly if a sex pheromone actually acts as an aggregation pheromone, which may change the whole strategy for the application of pheromone in a pest management system”. Taking this suggestion, applying thorough investigation as pheromone use expands will improve our ability to avoid unexpected negative outcomes while turning pheromones that elicit attraction from both sexes to our advantage by using them in methods such as attract-and-kill or mass trapping.

## Figures and Tables

**Table 1:** Behavioral responses of females to conspecific sex pheromones.

Family	Species	Study Type	Response ^1^	Dose Tested	Reference
Lepidoptera ^2^: Tortricidae	*Adoxophyes orana* (Fischer von Röslerstamm)	Observation chamber	Y	0.01–100 µg	[25]
	*Adoxophyes honmai* (Yasuda)	Observation chamber	Y	0.01–100 µg	[25]
	*Argyrotaenia velutinana* (Walker)	Observation chamber	Y		[26]
	*Choristoneura fumiferana* (Clemens)	Observation chamber	Y	2 mg	[27]
		Observation chamber	Y	1 pg–1 mg	[17]
		Faraday cage	Y	(100–200 ng/h)	[18]
	*Choristoneura rosaceana* (Harris)	Observation chamber	Y		[26]
		Observation chamber	N	255 mg	[28]
				(0.04 mg/h)	
		Observation chamber	Y	0.002–0.05 mg	[23]
		Flight mill			
	*Cydia fagiglandana* (Zeller)	Observation chamber	Y	10 µg blend	[29]
	*Cydia pomonella* (Linnaeus)	Observation chamber	Y	100 µg	[30]
				(0.93 µg/h)	
		Observation chamber	N	109 mg	[28]
				(0.02 mg/h)	
	*Cydia splendana* (Hübner)	Observation chamber	Y	10 µg blend	[29]
	*Eupoecilia ambiguella* (Hübner)	Observation chamber	N		[31]
	*Grapholita molesta* (Busck)	Observation chamber	Y		[32]
		Observation chamber	Y	238 µg	[28]
				(0.05 mg/h)	
		Observation chamber Flight mill	Y	0.002–0.05 mg	[23]
	*Homona magnanima* (Diakonoff)	Observation chamber	Y	0.01–100 µg	[25]
	*Lobesia botrana* (Denis and Schiffermüller)	Observation chamber	N		[31]
	*Pandemis limitata* (Robinson)	Observation chamber	N		[33]
	*Pandemis pyrusana* (Kearfott)	Observation chamber	Y	255 mg (0.04 mg/h)	[28]
Lepidoptera ^2^: Noctuidae	*Helicoverpa armigera* (Hübner)	Olfactometer	Y		[19]
	*Helicoverpa zea* (Boddie)	Olfactometer	Y		[19]
	*Heliothis subflexa* (Guenée)	Olfactometer	N		[34]
	*Pseudaletia adultera* (Schaus)	Observation chamber	Y	live ♀	[35]
	*Sesamia nonagrioides* (Lefebvre)	Olfactometer	Y		[36]
	*Spodoptera exigua* (Hübner)	Observation chamber	Y	10 µg	[37]
	*Spodoptera littoralis* (Boisduval)	Observation chamber	Y	0.1–5 mg	[20]
		Observation chamber	N		[31]
		Observation chamber	Y		[38]
	*Trichoplusia ni* (Hübner)	Field Trapping	Y		[7]
		Field Trapping	Y		[10]
Lepidoptera ^2^: Arctiidae	*Euplagia (Panaxia) quadripunctaria* (Poda)	Field observation	?		[6]
	*Utetheisa ornatrix* (Linnaeus)	Observation chamber	Y		[39,40]
		Observation chamber	Y		[41]
Lepidoptera ^2^: Sesiidae	*Melittia cucurbitae* (Harris)	Field trapping	Y		[42]
	*Vitacea polistiformis* (Harris)	Field observation	Y		[21]
Lepidoptera ^2^: Pyralidae	Ephestia *kuehniella* (Zeller)	Field trapping	Y		[22]
Coleoptera ^3^: Scarabaeidae	*Anomala orientalis* (Waterhouse)	Field trapping	Y		[43]
		Field observation	Y		[44]
	*Anomala rufocuprea* (Motschulsky)	Field trapping	?		[45]
		Field trapping	Y		[46]
	*Cotinis nitida* (Linnaeus)	Field trapping	Y		[47,48]
	*Holotrichia consanguinea* (Blanchard)	Field trapping and observation	Y		[49]
	*Holotrichia loochooana loochooana* (Sawada)	Field observation	Y	1–10 mg	[50]
		Field observation and experiment	Y	(800 ng/h)	[51]
	*Holotrichia serrata* (Fabricius)	Field observation	Y		[52]
	*Maladera (matrida)* *insanabilis* (Brenske)	Field observation	Y	live ♀	[53]
		Olfactometer	Y	live ♀	
		Field trapping	Y	live ♀/extract	
		Field trapping	Y	live ♀	[54]
Blattodea ^4^: Blattellidae	*Blattella germanica* (Linnaeus)	Observation and Olfactometer	Y		[55]
Diptera ^4^: Tephritidae	*Bactrocera (Dacus) oleae* (Rossi)	Field trapping	Y		[56]

^1^ “Y” = responded in some manner to pheromones; “N” = no observed response; “?” = unclear; ^2^ Lepidoptera with female-produced sex pheromones; ^3^ Scarabaeidae with female-produced sex pheromones not currently classified as aggregation pheromones in the literature; ^4^ Blattodea or Diptera with female-produced pheromones sometimes referred to as aggregation pheromones in the literature.

**Table 2:** Electro-physiological responses of females to conspecific sex pheromones.

Order: Family	Species	Test/Result ^1^		AD ^2^	Threshold ^3^	Reference
Lepidoptera: Tortricidae	*Adoxophyes orana* (Fischer von Röslerstamm)	SSR	+	+		[59]
	*Argyrotaenia velutinana* (Walker)	EAG	−	+		[60]
		EAG	+		≤2 µg	[26]
	*Choristoneura fumiferana* (Clemens)	EAG	+	+	<0.1 µg	[61]
		EAG	+		0.9 µg	[62]
	*Choristoneura rosaceana* (Harris)	EAG	+	+	≤2 µg	[26]
	*Cydia fagiglandana* (Zeller)	EAG	+	+	~0.1 µg	[29]
	*Cydia pomonella* (Linnaeus)	EAG	+	+	≤100 ng	[63]
		SSR	+			[64]
	*Cydia splendana* (Hübner)	EAG	+	+	~0.1 µg	[29]
	*Grapholita molesta* (Busck)	EAG	+	+	≤2 µg	[32]
	*Pandemis limitata* (Robinson)	EAG	+	+	≤10 µg	[33]
Lepidoptera: Noctuidae	*Agrotis segetum* (Denis and Schiffermüller)	EAG	−	−		[65]
		SSR	−			
	*Diparopsis castanea* (Hampson)	EAG	−	−		[66]
	*Helicoverpa armigera* (Hübner)	SSR	−	−		[67] ^4^
	*Helicoverpa zea* (Boddie)	EAG	−	−		[68]
	*Heliothis subflexa* (Guenée)	EAG	+	+		[69]
	*Heliothis virescens* (Fabricius)	EAG	+	+	≤10 µg	[70]
		SSR	+		1 µg	
		CR	+		≤12 µg	[71]
		EAG	+			[69]
		EAG	+			[15]
		SSR	+			
	*Pseudaletia adultera* (Schaus)	EAG	+	+	10 ng	[35]
	*Sesamia nonagrioides* (Lefebvre)	EAG	−	+		[72]
		EAG	+			[36]
	*Spodoptera exigua* (Hübner)	EAG	−	+		[73]
		EAG	+		0.01 µg	[37]
	*Spodoptera frugiperda* (Smith)	EAG	+	+	10 µg	[74]
	*Spodoptera littoralis* (Boisduval)	EAG	+	+		[66]
		EAG	+			[75]
		SSR	+			
		SSR	+		10–100 ng	[76]
	*Trichoplusia ni* (Hübner)	EAG	+	+	2–10 ng	[77]
		EAG	+		0.1 µg	[78]
		SSR	+		5 ng	[79]
Lepidoptera: Arctiidae	*Euplagia (Panaxia) quadripunctaria* (Poda)	EAG	+	+	0.01–1.0 µg	[6]
	*Utetheisa ornatrix* (Linnaeus)	SSR	+	+		[80]
Lepidoptera: Cossidae	*Coryphodema tristis* (Drury)	EAG	+	+		[81]
Lepidoptera: Sesiidae	*Melittia cucurbitae* (Harris)	EAG	+	+	0.1 µg	[42]
	*Vitacea polistiformis* (Harris)	EAG	+	+	1 µg	[82]
Lepidoptera: Yponomeuta	*Yponomeuta rorellus* (*rorrella*) (Hübner)	SSR	+	+		[83]
	*Yponomeuta vigintipunctatus* (Retzius)	SSR	+	+		[83]
Lepidoptera: Pyralidae	*Cactoblastis cactorum* (Berg)	EAG	+	+		[84]
		SSR	+			
Lepidoptera: Sphingidae	*Manduca sexta* (Linnaeus)	EAG	−	?		[85]
		EAG	−			[57]
		SSR	+			[58]
Lepidoptera: Geometridae	*Itame argillacearia* (Packard)	EAG	−	−		[86]
Lepidoptera: Bombycidae	*Bombyx mandarina* (Moore)	EAG	−	−		[87]
	*Bombyx mori* (Moore)	EAG	−	−		[4]
		SSR	−			[88]
Lepidoptera: Saturniidae	*Antheraea pernyi* (Guerin Meneville)	EAG	−	−		[4]
	*Antheraea polyphemus* (Cramer)	EAG	−	−		[89]
	*Attacus atlas* (Linnaeus)	EAG	−	−		[89]
	*Callosamia promethea* (Drury)	EAG	−	−		[4]
	*Eupackardia calleta* (Westwood)	EAG	−	−		[4]
	*Hyalophora cecropia* (Linnaeus)	EAG	−	−		[4]
Coleoptera: Scarabaeidae ^4^	*Anomala cuprea* (Hope)	EAG	−	+		[90]
		SSR	+			[91]
		SSR	+		10−100 ng	[92]
	*Anomala octiescostata* (Burmeister)	EAG	+	+		[93]
		EAG	+		0.1−10 µg	[94]
	*Anomala orientalis* (Waterhouse)	EAG	+	+	30 µg	[44]
	*Holotrichia serrata* (Fabricius)	EAG	+	+		[95]
Blattodea: Blattidae ^5^	*Periplaneta americana* (Linnaeus)	EAG	+	+		[96]
		GR	+			[97]

^1^ EAG = Electroantennagraph, SSR = single-sensillum recording, CR = cardiac response, GR = glomerular response; ^2^ AD = autodetection ability; “+” = verified; “−“ = not observed. (summary of all separate results for a species); ^3^ reported thresholds are for detection of major component unless noted; ^4^ Stranden and Mustaparta, unpublished, mentioned in [67]. ^5^ Scarabaeidae with female-produced sex pheromones not currently classified as aggregation pheromones in literature; ^6^ Blattodea with female-produced pheromones sometimes referred to as aggregation pheromones in literature.

**Table 3:** Expression of pheromone-binding and pheromone-receptor proteins in female antennae to conspecific sex pheromones.

Order: Family	Species	PBP ^1^ Present Female	PBP ^1^ Expression in Female ^2^	PR ^3^ Present Female	PR ^3,4^ Expression in Female ^2^	Reference
Lepidoptera: Tortricidae	*Cydia pomonella* (Linnaeus)			Y	moderate, similar to male	[105]
Lepidoptera: Noctuidae	*Agrotis ipsilon* (Hufnagel)	Y	moderate, similar to male			[106]
			moderate, ~33% of male			[107]
	*Agrotis segetum* (Denis + Schiffermüller)	Y	moderate	Y		[67]
			moderate, 30%–50% of male			[108]
			moderate, similar to male		low, less than male	[107]
	*Autographa gamma* (Linnaeus)	Y	moderate, similar to male			[109]
			moderate			[67]
	*Helicoverpa armigera* (Hübner)	Y	high	Y		[110]
			moderate-high, 37%–79% of male			[67]
					moderate, less than male	[111]
	*Helicoverpa zea* (Boddie)	Y	moderate, 40%–50% of male			[112]
	*Heliothis virescens* (Fabricius)	Y	low, weakly expressed	Y		[113]
			moderate, 40%–50% of male			[112]
			moderate			[67]
					not found	[114]
					found	[115]
	*Mamestra brassicae* (Linnaeus)	Y	unclear			[116]
			present			[117]
	*Mythimna separata* (Walker)	Y	high	?	not found (0:1)	[118]
	*Sesamia nonagrioides* (Lefebvre)	Y	abundant			[119]
			moderate, 15%–47% of male			[72]
	*Spodoptera exigua* (Hübner)	Y	moderate-high, 39%–73% of male			[120]
	*Spodoptera frugiperda* (Smith)	Y	moderate, 40%–50% of male			[112]
	*Spodoptera littoralis* (Boisduval)	Y	high	Y		[102] ^5^
			high			[67]
					moderate, 30%–90% of male	[121]
	*Spodoptera litura* (Fabricius)	Y	low, 2%–7% of male			[122]
Lepidoptera: Plutellidae	*Plutella xylostella* (Linnaeus)	Y	high	Y	not found (0:1)	[117]
					low, 1% of male	[123]
Lepidoptera: Pyralidae	*Chilo suppressalis* (Walker)	Y	moderate, similar to male	Y		[124]
					moderate, similar to male	[125]
	*Orthaga achatina* (Butler)	Y	moderate, ~25%–33%			[111]
Lepidoptera: Crambidae	*Cnaphalocrocis medinalis* (Guenée)	Y	low, 1%–32%			[126]
	*Diaphania indica* (Saunders)	Y	moderate	?	not found (0:1)	[118]
Lepidoptera: Sphingidae	*Manduca sexta* (Linnaeus)	Y	not detected	N		[100]
			low, 14% of male			[101]
			low			[127]
			low			[128]
			present			[129]
					not found (0:2)	[130]
Lepidoptera: Bombycidae	*Bombyx mori* (Moore)	Y	low	Y		[109]
			rare			[102]
					not found (0:1)	[131]
					found (4:6)	[103]
			rare			[132]
			low			[133]
Lepidoptera: Saturniidae	*Antheraea pernyi* (Guerin Meneville)	Y	not detected			[100]
			not detected			[134]
			rare			[102]
	*Antheraea polyphemus* (Cramer)	Y	not detected	N		[100]
			not detected			[109]
			very rare			[135]
			rare			[102]
					extremely low	[104]
	*Hyalophora cecropia* (Linnaeus)	N	not detected			[100]
Coleoptera: Scarabaeidae	*Anomala cuprea* (Hope)	Y	moderate, similar to male			[94]
	*Anomala octiescostata* (Burmeister)	Y	moderate, similar to male			[94]

^1^ PBP = pheromone-binding proteins; relative estimates include PBP proteins or PBP mRNA; ^2^ when no numerical estimate was provided, statements such as “levels similar to males” were categorized as “moderate”; ^3^ PR = pheromone-receptor proteins; relative estimates include PR proteins or PR mRNA; ^4^ numbers in parenthesis are ratios of no. PR proteins “found” *versus* no. PR proteins “tested for” (found: tested for); ^5^ Steinbrecht, unpublished, mentioned in [102].

**Table 4:** Summary of autodetection in females.

Order: Family	Species	Behavioral Response ^2^	Electro-Physiological Response ^1^	PBP/PR Presence ^3,4^	Expression of Antennal Detection Proteins ^5^	
					PBP ^3^	PR ^3^
Lepidoptera: Tortricidae	*Adoxophyes orana*	Y	+	+		
	*Adoxophyes honmai*	Y				
	*Argyrotaenia velutinana*	Y	+	+		
	*Choristoneura fumiferana*	Y	+	+		
	*Choristoneura rosaceana*	Y	+	+		
	*Cydia fagiglandana*	Y	+	+		
	*Cydia pomonella* ^6^	Y	+	+	•	•
	*Cydia splendana*	Y	+	+		
	*Eupoecilia ambiguella*	N				
	*Grapholita molesta*	Y	+	+		
	*Homona magnanima*	Y				
	*Lobesia botrana*	N				
	*Pandemis limitata*	N	+	+		
	*Pandemis pyrusana*	Y				
Lepidoptera: Noctuidae	*Agrotis ipsilon*			+	**〇**	
	*Agrotis segetum* ^6^		-	+	•	Ø
	*Autographa gamma*			+	**〇**	
	*Diparopsis castanea*		-			
	*Helicoverpa armigera*	Y	-	+	•	**〇**
	*Helicoverpa zea* ^6^	Y	-	+	**〇**	
	*Heliothis subflexa*	N	+	+		
	*Heliothis virescens* ^6^		+	+	**〇**	**〇**
	*Mamestra brassicae*			+	Ø	
	*Mythimna separate*			+	•	?
	*Pseudaletia adultera*	Y	+			
	*Sesamia nonagrioides* ^6^	Y	+	+	**〇**	
	*Spodoptera exigua* ^6^	Y	+	+	•	
	*Spodoptera frugiperda*		+	+	**〇**	
	*Spodoptera littoralis* ^6^	Y	+	+	•	**〇**
	*Spodoptera litura*			+	Ø	
	*Trichoplusia ni*	Y	+	+		
Lepidoptera: Arctiidae	*Euplagia quadripunctaria*	?	+	+		
	*Utetheisa ornatrix*	Y	+	+		
Lepidoptera: Cossidae	*Coryphodema tristis*		+			
Lepidoptera: Sesiidae	*Melittia cucurbitae*	Y	+	+		
	*Vitacea polistiformis*	Y	+	+		
Lepidoptera: Yponomeuta	*Yponomeuta rorellus*		+	+		
	*Yponomeuta vigintipunctatus*		+	+		
Lepidoptera: Plutellidae	*Plutella xylostella*			+	•	Ø
Lepidoptera: Pyralidae	*Cactoblastis cactorum*		+	+		
	*Chilo suppressalis*			+	**〇**	**〇**
	*Ephestia kuehniella*	Y				
	*Orthaga achatina*			+	**〇**	
Lepidoptera: Crambidae	*Cnaphalocrocis medinalis*			+	Ø	
	*Diaphania indica*			+	**〇**	?
Lepidoptera: Sphingidae	*Manduca sexta* ^6^		+ *	+	Ø	X
Lepidoptera: Geometridae	*Itame argillacearia*		-			
Lepidoptera: Bombycidae	*Bombyx mandarina*		-			
	*Bombyx mori* ^6^		-	+	Ø	Ø
Lepidoptera: Saturniidae	*Antheraea pernyi* ^6^		-	+	Ø	
	*Antheraea Polyphemus* ^6^		-	+	Ø	X
	*Attacus atlas*		-			
	*Callosamia promethean*		-			
	*Eupackardia calleta*		-			
	*Hyalophora cecropia* ^6^		-		X	
Coleoptera: Scarabaeidae	*Anomala cuprea* ^6^		+	+	**〇**	
	*Anomala octiescostata* ^6^		+	+	**〇**	
	*Anomala orientalis*	Y	+	+		
	*Anomala rufocuprea*	Y				
	*Cotinis nitida*	Y				
	*Holotrichia consanguinea*	Y				
	*Holotrichia loochooana loochooana*	Y				
	*Holotrichia serrata*	Y	+	+		
	*Maladera insanabilis*	Y				
Blattodea:	*Periplaneta Americana*		+	+		
	*Blattella germanica*	Y				
Diptera: Tephritidae	*Bactrocera oleae*	Y	+	+		

^1^ Electro-physiological response: (+) verified, (−) not detected, (?) unclear, (+*) response to minor component only; ^2^ behavior response: (Y) verified, (N) not observed, (?) unclear; ^3^ PBP = Pheromone-Binding Protein, PR = Pheromone-Receptor Protein; ^4^ PBP/PR Presence: (+) confirmed present or assumed from EAG/SSR response, (−) undetected; ^5^ PBP/PR Expression: • = high (>66%–100%), **〇** = moderate (>33%–66%), Ø = low (0%–33%), X = not detected, ? = undetermined; ^6^ species used in Figure 2A–C.

## Abbreviations

The following abbreviations are used in this manuscript:

EAG	Electroantennogram
SSR	Single-sensillum recording
CR	Cardiac response
GR	Glomerular response
PBP	Pheromone-binding protein
PR	Pheromone receptor protein
